# Anomaly Detection and Objective Security Evaluation Using Autoencoder, Isolation Forest, and Multi-Criteria Decision Methods

**DOI:** 10.3390/s25196250

**Published:** 2025-10-09

**Authors:** Hongbin Zhang, Haibin Zhang

**Affiliations:** School of Cyber Engineering, Xidian University, Xi’an 710071, China; zhanghongbinw@126.com

**Keywords:** cybersecurity testing, autoencoder (AE), isolation forest (IF), evaluation system

## Abstract

With the rapid development of cybersecurity technologies, cybersecurity testing has played an increasingly critical role in scientific research, new technology validation, system performance evaluation, and talent development. A central challenge in this domain lies in efficiently and rapidly constructing testing environments while ensuring the reliability and reproducibility of test results. To address this issue, this paper proposes an integrated evaluation method specifically designed for cybersecurity testing, combining anomaly detection and predictive analysis techniques. The method first employs an autoencoder (AE) to perform dimensionality reduction on the raw data collected from a testbed environment, followed by anomaly detection using the Isolation Forest (IF) algorithm. To assess the impact of cyberattacks on the stability of the testbed system, the steady-state data of the environment was treated as the ideal reference. The degree of disruption was then quantified by calculating the Euclidean distance between the dimensionally reduced experimental data and the reference state. Finally, a specific case study was conducted to validate the feasibility and effectiveness of the proposed method, and a percentage-based scoring mechanism was introduced to quantitatively evaluate the security level of the system.

## 1. Introduction

In cybersecurity research, the construction and application of cyber ranges have become critical tools for testing and validating security technologies. Cyber range targets—virtual or physical assets deployed in simulated or real environments—are used to evaluate system vulnerabilities and the effectiveness of defensive mechanisms [1,2]. The diversity of these targets is essential for enhancing defensive capabilities, simulating complex attack scenarios, and improving the reliability of evaluation results.

This diversity spans domains such as industrial control systems (ICSs), financial systems, military networks, and the Internet of Things (IoT) [3]. For instance, IoT targets replicate device interactions in smart environments [4], while ICSs ranges simulate industrial network components and protocols (e.g., SCADA, PLC, and HMI) using hybrid platforms [5,6], enabling vulnerability discovery and the evaluation of control mechanisms [7,8,9].

While such diversity enhances the realism of simulations, it also increases evaluation complexity—particularly in the fusion of heterogeneous data [10,11,12]. To address this, researchers have developed data fusion algorithms that support unified analysis across data formats, timestamps, and domains, thereby improving the accuracy of cybersecurity assessments [13,14,15].

Building on this context and experimental insights, this paper proposes a quantitative security assessment system for cyber range targets, integrating anomaly detection with multi-objective decision making. The proposed method consists of the following core steps.

Dimensionality Reduction and Feature Construction: An autoencoder reduces steady-state data, and temporal features are extracted using time windows.

Anomaly Detection: Data from attack periods are similarly reduced and used to train an Isolation Forest for identifying anomalies.

Multi-Objective Assessment: Anomaly scores are used to evaluate deviations from normal operational states, supporting comprehensive ranking across multiple criteria.

## 2. Related Work

With the accelerated development of informationization in industrial control systems (ICSs), the integration of industrialization and informatization has reached unprecedented levels. Traditional ICSs were initially designed and deployed using proprietary hardware, software, and industrial control protocols, with little consideration for system openness. As ICSs become increasingly interconnected and open, they are facing a growing number of security threats. The security assessment of ICSs has emerged as a key research topic, as it provides insight into the system’s security posture, identifies vulnerabilities, and serves as a proactive defense mechanism against potential threats [16,17,18,19].

Numerous researchers have explored various methods for ICS security assessment. One representative approach is the Bayesian network (BN), which integrates probability theory and graph theory to model and analyze security risks under uncertainty. For instance, Wang et al. [20] utilized a Bayesian network model to represent multiple risk-related factors, combining probabilistic methods with expert knowledge. This method allows for both global and local risk assessments within the network. However, a major limitation lies in the assignment of values to input nodes—typically representing the likelihood of device compromise—which remains highly subjective and lacks standardized quantitative metrics.

Another widely adopted technique is the Analytic Hierarchy Process (AHP). The AHP constructs hierarchical structures and assigns weights to evaluate system security. Bian et al. [21] assessed cybersecurity posture from the perspectives of risk, service, and public factors. Lu Huikang et al. [22] developed a security evaluation framework based on the NIST 800-82 and IEC 62443 standards. While AHP provides a clear and logical structure, its effectiveness heavily relies on expert judgment, particularly in translating the likelihood of device compromise into relative weight values. To address the inherent subjectivity, some researchers have combined the AHP with fuzzy comprehensive evaluation methods [23,24], aiming to better handle uncertainty and improve robustness. However, these hybrid approaches still lack solid data-driven support and remain dependent on expert knowledge. To mitigate this limitation, realistic industrial control testbeds and datasets have been developed, such as the Secure Water Treatment (SWaT) dataset [25], which provides comprehensive operational and attack data from a real-world water treatment plant and serves as a valuable resource for reproducible experiments and objective evaluations.

In summary, existing ICS security assessment methods have laid a solid theoretical and practical foundation. Nonetheless, challenges, such as subjectivity, lack of real-time responsiveness, and limited objective quantification, persist. These limitations highlight the need for more data-driven, intelligent, and automated assessment approaches.

## 3. Methodology

**Figure 1:**
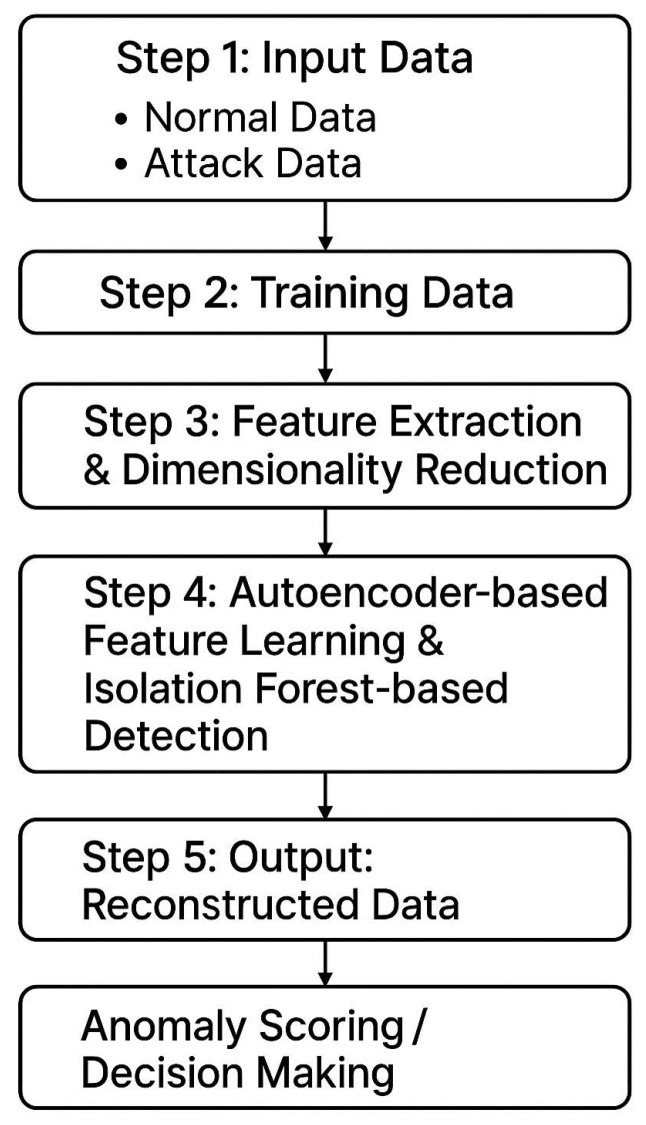
System flow.

The overall workflow of the proposed system is illustrated in the figure below (Figure 1). This method used two types of datasets as training samples: one representing system parameters during stable operation, and the other representing parameters collected after a cyberattack. First, an autoencoder model was trained on the stable operation data, with a time-window mechanism incorporated to capture the temporal dynamics of system behavior. Then, data collected during attack periods were passed through the trained autoencoder for dimensionality reduction. The resulting low-dimensional features were used to train an Isolation Forest model for anomaly detection.

In parallel, a relational matrix was constructed using the dimensionally reduced stable data, and it is used for analysis via the TOPSIS method (Technique for Order Preference by Similarity to Ideal Solution). An additional column representing the anomaly detection distance was appended to the matrix to refine the overall evaluation score. To ensure the objectivity and scientific rigor of the assessment, the Entropy Weight Method (EWM) was applied to assign appropriate weights to each feature dimension. These components together form the complete system model.

In practical deployment, real-time system data is fed into the model. The data is first processed by the autoencoder for dimensionality reduction and then simultaneously passed to both the Isolation Forest model and the relational matrix. The anomaly detection distance, calculated by the Isolation Forest, is appended to the matrix. The EWM is then used to determine the weights for each dimension, and the final system evaluation score is calculated based on the weighted TOPSIS analysis.

The following subsections of this chapter provide a detailed explanation of each component in the proposed methodology.

### 3.1. Autoencoder

**Figure 2:**
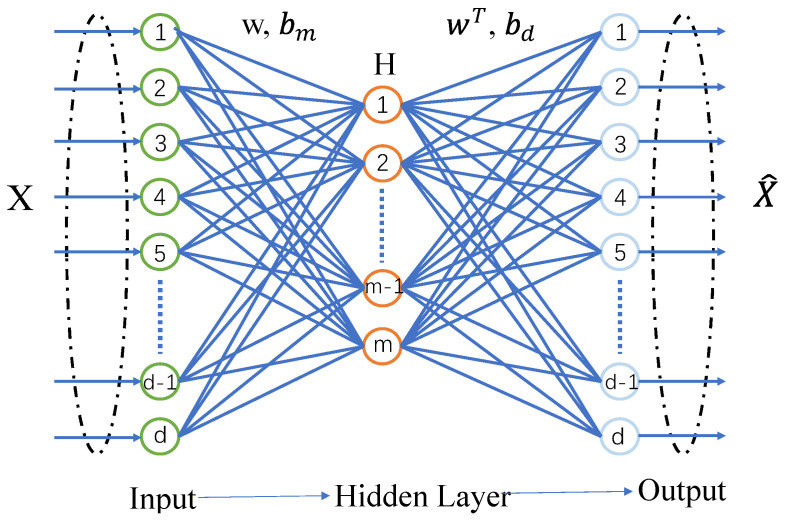
Autoencoder architectures.

An AE typically consists of two components: an encoder and a decoder. The encoder converts complex high-dimensional data into simplified low-dimensional representations, enabling efficient data compression and dimensionality reduction. The decoder, in turn, converts the abstract representations back into the expected output, thereby reconstructing the input samples. The structure of the AE is illustrated in Figure 2.

Similar to fully connected neural networks, autoencoders (AEs) also use fully connected nodes but follow an unsupervised learning approach, with both inputs and outputs being unlabeled. The goal of an AE is to learn the intrinsic structure of the data and extract generalized features without relying on label information.

The training process consists of two stages: encoding and decoding. The encoder maps input data to a latent representation, while the decoder reconstructs the original input. Network parameters are optimized by minimizing the reconstruction error, resulting in an abstract representation of the input.

Given an input sample $X=R^{d*n}$, with the weight matrix *W* between the input and encoding layers, encoding bias $b_{m}$, decoding bias $b_{d}$, and activation function g(), the encoding step is defined as follows:

(1) $$H=g(WX+b_{m}).$$

Next, the decoder decodes the encoded features to obtain the reconstructed input sample $\overset{\wedge }{X}$. Given the encoding *H*, $\overset{\wedge }{X}$ can also be viewed as a prediction of *X*, having the same dimensions as *X*. The decoding process is similar to the encoding process.

(2) $$\overset{\wedge }{X}=g\left(W^{T}H+b_{d}\right).$$

The training objective of an AE is to minimize the value of the loss function.

(3) $$arg\min_{W,b}\left(W,b\right).$$

In the system proposed in this paper, we utilized the mean squared error (MSE) as the loss function. MSE is a commonly used loss function in regression tasks, designed to measure the discrepancy between the model’s predicted values and the actual values. Specifically, MSE reflects the model’s prediction accuracy by calculating the squared difference between the predicted and true values, and it then averages these squared differences across all data points. The formula for MSE is as follows:

(4) $$MSE=\frac{1}{n}\sum_{i=1}^{n}{\left(y_{i}-\overset{\wedge }{y_{i}}\right)}^{2}.$$

Here, *n* denotes the number of samples, $y_{i}$ is the actual value of the *i*-th sample, and $\overset{\wedge }{y_{i}}$ represents the predicted value of the *i*-th sample.

AEs commonly utilize optimization algorithms, such as stochastic gradient methods, for error backpropagation and network parameter adjustment. Through iterative fine tuning, the reconstruction error function is gradually minimized, allowing the AE to learn key abstract features from the sample data. When using the gradient descent algorithm, assuming the learning rate is η, the update rules for the connection weights and biases of the AE are as follows:

(5) $$W=W-\eta \frac{\partial J(W,b)}{\partial W},$$

(6) $$b=b-\eta \frac{\partial J(W,b)}{\partial b}.$$

We cascaded AEs to construct a stacked autoencoder, which employs layer-wise greedy training. In this approach, the hidden layer output of the preceding AE is used as the input for the subsequent AE, enabling hierarchical feature extraction. This method ensures that the final extracted features are more representative and typically of lower dimensionality [26]. Stacked autoencoders usually have a symmetrical multi-hidden-layer structure corresponding to the encoding and decoding processes. However, when applied to classification or regression problems, the decoding part is generally discarded, and the final encoding is used for classification [27] or regression [28].

Compared to commonly used unsupervised learning algorithms like Principal Component Analysis (PCA) and Independent Component Analysis (ICA), autoencoders (AEs) perform feature extraction by weighted integration, transforming features into higher-level abstractions that are both lower in dimensionality and more representative. AEs effectively leverage important information from secondary features rather than simply discarding them. As a result, the abstract features extracted or dimensionality reduced by AEs are more conducive to classification and regression tasks. In comparison to Restricted Boltzmann Machines (RBMs), AEs do not require the sampling operations involved in contrastive divergence, leading to shorter training times. Additionally, compared to Self-Organizing Maps (SOMs), AEs can map feature spaces of arbitrary dimensions by adjusting the number of hidden layer nodes.

Therefore, we employed AEs to perform dimensionality reduction on the target data. The target data has high dimensionality and exhibits significant nonlinearity. Additionally, certain data points may remain unchanged over extended periods, but when they do change, they can have a substantial impact on the system. In some dimensionality reduction methods, these critical values might be directly overlooked.

### 3.2. Isolation Forest

The IF algorithm [29] is an outlier detection method based on tree models. Its function lies in identifying anomalous data within a dataset by using an “isolation” strategy, making it highly effective for applications in target detection.

**Figure 3:**
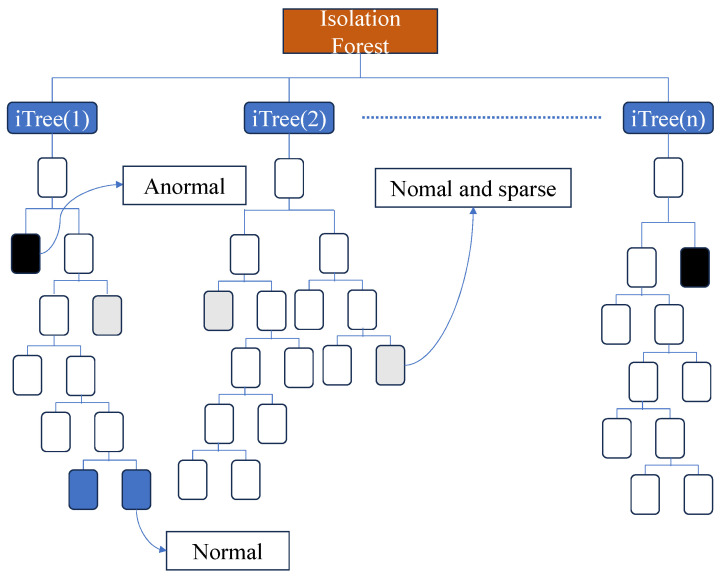
Isolation Forest model.

In IF, anomalous data points are defined as outliers that are easily “isolated.” These outliers typically exhibit sparse distributions and are located at a certain distance from regions with densely distributed points. For the distribution of the dimensionality-reduced sample data, well-performing data points can be seen as densely distributed with a high probability, while sparsely distributed data points are considered low-probability and anomalous. Therefore, as illustrated in Figure 3, IF identifies outliers by recursively and randomly partitioning the dataset multiple times until no further partitioning is possible. In this approach, “isolating” dense clusters requires many partitions, whereas sparsely distributed points can be “isolated” with only a few partitions.

IF incorporates the concept of ensemble learning by generating multiple “tree-structured” learners and synthesizing the outputs of each learner to achieve a more accurate final result. The construction of each learner (ITree) is based on a completely random generation process.

1. When initially constructing an Isolation Tree (ITree), *m* samples are uniformly drawn from *n* groups of sampled data to form a subsample.
2. Within the subsample, a feature dimension is randomly selected. A value *k* is then randomly chosen within the range of this feature’s values (between the minimum and maximum). The samples are categorized into two groups based on this value: those with values less than *k* are allocated to the left branch, while those with values no less than *k* are placed in the right branch.
3. The process in Step 2 is then repeated on the newly generated left and right data subsets. The splitting continues until either the data can no longer be divided or the number of splits reaches $log_{2}k$, at which point the splitting operation stops.

After constructing *t* Isolation Trees (ITrees), each sample data point *x* traverses every ITree, and the path length h(x) is calculated for each traversal. The path length h(x) represents the number of edges traversed from the root node to the leaf node. Finally, h(x) is substituted into Equation (7) to calculate the anomaly score of each data point. The anomaly score of *x* is defined as follows:

(7) $$S\left(x,n\right)=2^{-\frac{E(h(x))}{c(n)}},$$

where S(x,n) represents the anomaly score for each sampled data point, while E(h(x)) is the expected path length of sample *x* across all Isolation Trees (ITrees).

(8) $$C\left(n\right)=2H\left(n-1\right)-\frac{2(n-1)}{n},$$

where c(n) represents the average path length for a given sample size *n*, used to normalize the sample’s path length h(x); and the harmonic number H(i) can be estimated as ln(i)+γ, where γ is the Euler–Mascheroni constant.

The value of *S* ranges from 0 to 1. The closer *S* is to 1, the higher the likelihood that the data is considered anomalous. When S<0.5, the data can be judged as non-anomalous. If *S* is around 0.5, the data is not deemed to have clear anomalous characteristics and may require further observation.

### 3.3. TOPSIS

The TOPSIS (Technique for Order of Preference by Similarity to Ideal Solution) method is a distance-based multi-attribute decision-making technique that ranks alternatives by calculating their distances from an ideal solution and a negative ideal solution. The fundamental principle of TOPSIS is to select an optimal solution that is closest to the positive ideal solution and farthest from the negative ideal solution.

In the TOPSIS method, the first step is to construct a relational matrix for evaluation. Let there be *m* alternatives $A_{1},A_{2},\cdots ,A_{m}$, and *n* decision attributes $C_{1},C_{2},\cdots ,C_{m}$. The values of each alternative with respect to each attribute can be represented in the decision matrix *X* as follows:

(9) $$X=\left[\begin{array}{cccc} x_{11} & x_{12} & \ldots & x_{1n}\\ x_{21} & x_{22} & \ldots & x_{2n}\\ \vdots & \vdots & \ddots & \vdots \\ x_{m1} & x_{m1} & \ldots & x_{mn}. \end{array}\right]$$

Here, $x_{ij}$ represents the value of alternative $A_{i}$ under attribute $C_{j}$.

After constructing the decision matrix, it is necessary to eliminate the dimensional differences between attributes by standardizing the matrix. Since the parameter values in industrial control systems typically fall within a defined range (e.g., 0 to 100), and the steady-state values are also within this range, we adopted min-max normalization. This method scales different parameters to the same range, making it easier to compare their performance under a unified standard.

The formula for min-max normalization is as follows:

(10) $$r_{ij}=\frac{x_{ij}-x_{j}^{min}}{x_{j}^{max}-x_{j}^{min}},$$

where $r_{ij}$ is the normalized value of alternative $A_{i}$ under attribute $C_{j}$, $x_{ij}$ is the original value, and $x_{j}^{min}$ and $x_{j}^{max}$ are the minimum and maximum values of attribute $C_{j}$, respectively.

This formula scales all parameter values to the range [0, 1], ensuring that all parameters can be compared on the same scale.

Thus, we obtained the normalized matrix *R*:

(11) $$R=\left[\begin{array}{cccc} r_{11} & r_{12} & \ldots & r_{1n}\\ r_{21} & r_{22} & \ldots & r_{2n}\\ \vdots & \vdots & \ddots & \vdots \\ r_{m1} & r_{m1} & \ldots & r_{mn} \end{array}\right],$$

where $r_{ij}$ represents the normalized value of alternative $A_{i}$ under attribute $C_{j}$.

Since different attributes may have varying levels of importance, it is necessary to introduce a weight vector $w=(w_{1},w_{2},\cdots ,w_{n})$, where $\sum_{j=1}^{n}w_{j}=1$. The normalized matrix is then weighted using the following formula:

(12) $$v_{ij}=r_{ij}\cdot w_{j},$$

where $v_{ij}$ represents the weighted value of alternative $A_{i}$ under attribute $C_{j}$, $r_{ij}$ is the normalized value, and $w_{j}$ is the weight assigned to attribute $C_{j}$.

Thus, we obtained the weighted normalized matrix *V*:

(13) $$V=\left[\begin{array}{cccc} v_{11} & v_{12} & \ldots & v_{1n}\\ v_{21} & v_{22} & \ldots & v_{2n}\\ \vdots & \vdots & \ddots & \vdots \\ v_{m1} & v_{m1} & \ldots & v_{mn} \end{array}\right].$$

The values of the weights *w* are determined using the Entropy Weight Method (EWM), which objectively assigns weights to each attribute based on the amount of information (or variability) the attribute contains. The fundamental idea behind EWM is that entropy reflects the uncertainty or fluctuation of the data—attributes with greater variability carry more information and should be assigned higher weights. The detailed derivation process is as follows.

Using the normalized matrix, the proportion $p_{ij}$ of alternative $A_{i}$ under attribute $C_{j}$ is calculated as follows:

(14) $$p_{ij}=\frac{r_{ij}}{\sum_{i=1}^{m}r_{ij}},$$

where $r_{ij}$ is the normalized value, and $p_{ij}$ represents the relative importance of alternative $A_{i}$ under attribute $C_{j}$.

The entropy $e_{j}$ for attribute $C_{j}$ is computed using the following formula:

(15) $$e_{j}=-k\sum_{i=1}^{m}p_{ij}ln\left(p_{ij}\right),$$

where $k=\frac{1}{ln(m)}$ is a normalization constant to ensure that the entropy value $e_{j}$ lies between [0,1]. When $p_{ij}=0$, ln is defined as 0 to avoid logarithmic issues.

The entropy $e_{j}$ reflects the uncertainty of attribute $C_{j}$ across all alternatives. A higher entropy indicates more uniform distribution and greater uncertainty, while lower entropy suggests that the attribute contains more useful information.

To assess the amount of useful information an attribute provides, we computed the degree of diversification (or dispersion) for each attribute $C_{j}$:

(16) $$d_{j}=1-e_{j}.$$

The higher the diversification $d_{j}$, the more the attribute $C_{j}$ contributes to distinguishing between alternatives, meaning it should be assigned a greater weight.

Finally, the weights $w_{j}$ for each attribute $C_{j}$ are determined by normalizing the degree of diversification:

(17) $$w_{j}=\frac{d_{j}}{\sum_{j=1}^{n}d_{j}}.$$

Next, we determined the ideal and negative ideal solutions based on the anomaly distances calculated using the Isolation Forest model. Specifically, the point with the smallest anomaly distance was considered the ideal solution, while the point with the largest anomaly distance was regarded as the negative ideal solution.

After determining the ideal and negative ideal solutions, we calculated the Euclidean distance between each point and the ideal solution $A^{+}$ and the negative ideal solution $A^{-}$, i.e., The distance to the ideal solution $D_{i}^{+}$, as follows:

(18) $$D_{i}^{+}=\sqrt{\sum_{j=1}^{m}{\left(v_{ij}-A_{j}^{+}\right)}^{2}}.$$

The distance to the negative ideal solution $D_{i}^{-}$ is as follows:

(19) $$D_{i}^{-}=\sqrt{\sum_{j=1}^{m}{\left(v_{ij}-A_{j}^{-}\right)}^{2}}.$$

Using the distances to the ideal and negative ideal solutions, we calculated the relative closeness $C_{i}$ for each option. The formula is as follows:

(20) $$C_{i}=\frac{D_{i}^{-}}{D_{i}^{-}+D_{i}^{+}}*100.$$

The closer the relative closeness $C_{i}$ is to 100, the closer option *i* is to the ideal solution and the farther it is from the negative ideal solution, which results in a higher ranking. The value of $C_{i}$ also represents the current system score.

## 4. Results

In this paper, we used the SWaT (Secure Water Treatment) dataset as a case study, employing both normal operation data and labeled attack data from the SWaT system as training samples. Based on this data, we separately constructed an autoencoder model and an Isolation Forest model. Finally, the system’s state was evaluated in real time using a test dataset. It is worth noting that the test dataset includes attack behaviors during specific time periods, which are used to assess the effectiveness of the models in detecting anomalies.

The SWaT dataset originates from a small-scale, fully operational water treatment testbed developed by the Singapore University of Technology and Design. It is designed to simulate real-world industrial water treatment processes. The testbed consists of six physical stages, replicating the entire water treatment process from raw water intake to final water output.

The dataset records high-frequency data from sensors and actuators under both normal operations and cyber-physical attacks. The attack scenarios cover a wide spectrum of threats at both the network and physical levels, including sensor data manipulation and interference with actuator control signals. Each data point is carefully labeled as either normal or anomalous, supporting supervised learning and model validation.

The SWaT dataset serves as a vital resource for research in industrial control system (ICS) security, particularly in anomaly and intrusion detection. Owing to its high fidelity and realism, it has been extensively utilized in academic studies, with numerous papers relying on it to develop and benchmark security algorithms.

The experimental platform used in this study features dual Intel E5-2673V4 processors and dual NVIDIA RTX 3090 GPUs. The system operates on Ubuntu 24.04. The primary objective of testing was to evaluate the system’s anomaly detection performance and to determine whether the model’s scoring aligns with actual system behavior during attack scenarios.

### 4.1. Anomaly Detection

In this section, we focus on evaluating the system’s anomaly detection capabilities, aiming to verify the effectiveness and reliability of dimensionality-reduced data in the assessment process. To this end, we designed a series of experiments: first, we compared the original data, the data reduced via PCA, and the data reduced via an autoencoder, and we then visualized their distributions in a three-dimensional plot to observe the separation effect of each dataset.

**Figure 4:**
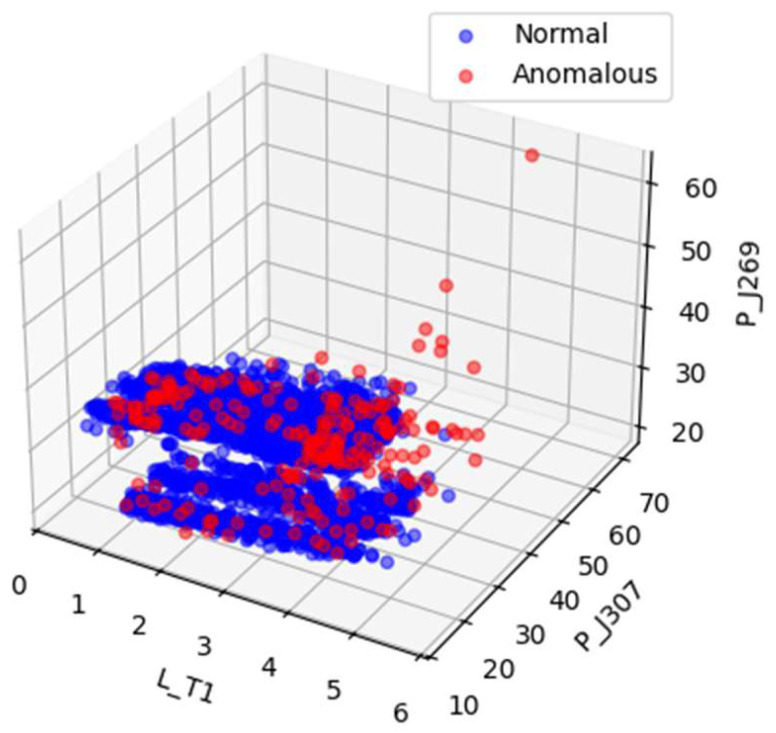
Visualizations of random subsets of the SWaT normalized raw data.

**Figure 5:**
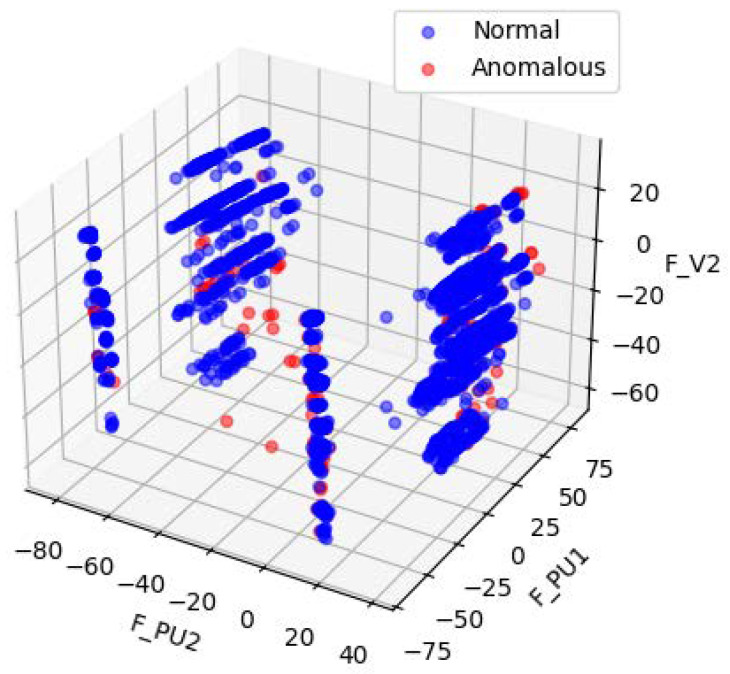
Visualizations of the SWaT PCA-processed data.

**Figure 6:**
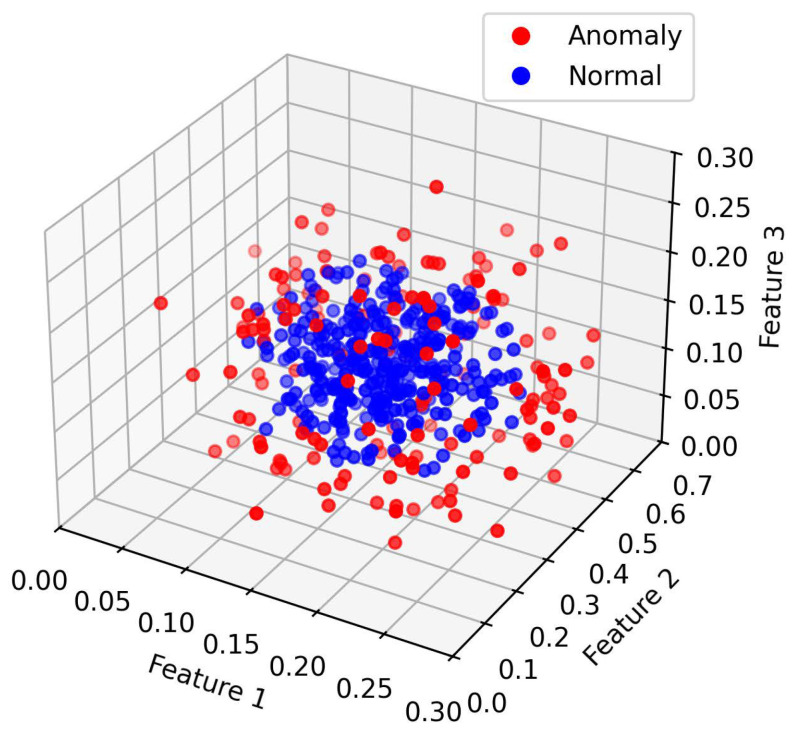
Visualizations of the SWaT AE-processed data.

Figure 4 and Figure 5 illustrates the data distribution after dimensionality reduction to three dimensions using Principal Component Analysis (PCA), while Figure 4 shows the distribution based on a random selection of three features from the original normalized dataset. Figure 6 presents the data distribution after being reduced to three dimensions using an autoencoder.

In the original dataset, outliers and normal points appear relatively clustered, with no evident trend of separation. After PCA-based dimensionality reduction, outliers begin to shift toward the periphery of the distribution. While some degree of separation between outliers and normal data becomes visible, the boundary remains ambiguous, indicating that the effectiveness of PCA in distinguishing anomalies is limited. In contrast, dimensionality reduction using the autoencoder results in a more distinct separation between outliers and normal data, demonstrating superior discriminative capability.

In the following experimental section, we compare the model’s performance across different time windows and varying numbers of Isolation Forest trees. The comparison includes metrics such as the F1 score, accuracy, and recall. The objective of this comparison was to evaluate the impact of these parameters on the model’s ability to detect anomalies.

**Figure 7:**
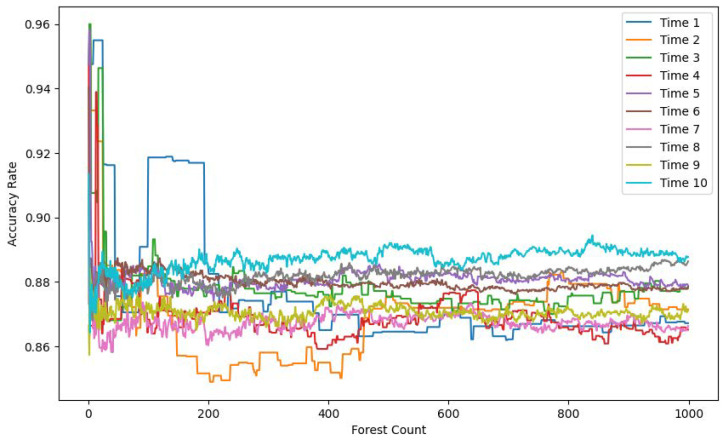
Accuracy rate comparison.

**Figure 8:**
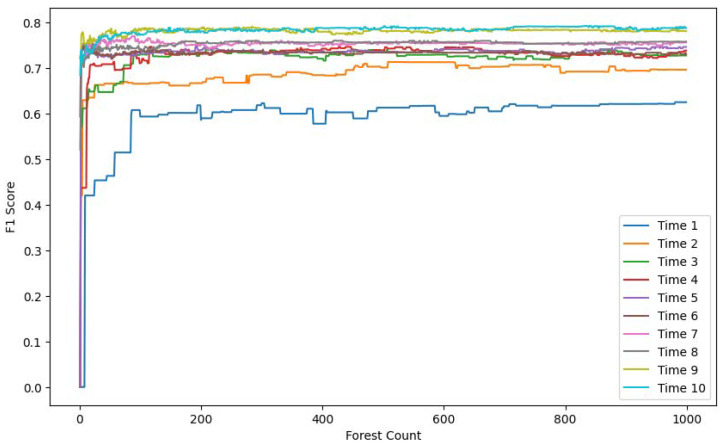
F1 score comparison.

**Figure 9:**
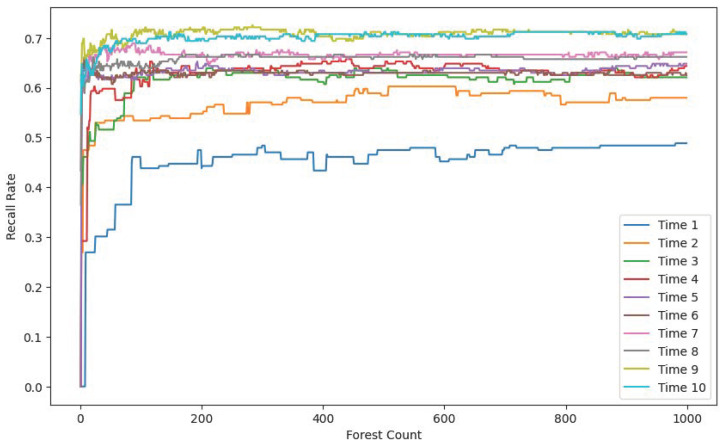
Recall rate comparison.

Figure 7, Figure 8 and Figure 9 each display ten lines, with each line representing a different time window size. The horizontal axis indicates the number of trees in the Isolation Forest model, while the vertical axes correspond to the accuracy, F1 score, and recall, respectively.

As the number of trees increases, both the F1 score and recall improve and stabilize around 200 trees. In contrast, the accuracy shows a gradual decline, also stabilizing around 200 trees. This drop in accuracy is due to the model’s limited ability to detect anomalies when using a small number of trees. Given the severe imbalance between normal and anomalous samples in the test set, a smaller number of trees leads to the misclassification of anomalies as normal instances, resulting in artificially high and misleading accuracy. With more trees, the model’s actual detection capability improves, as reflected in the rising F1 score and recall.

The results also demonstrate that model performance varies significantly with time window size. A window size of 1 means no temporal context is included. As the window size increases, the recall, F1 score, and accuracy all improve, eventually plateauing at a window size of 10.

As shown in Table 1, the proposed model significantly outperforms other comparable algorithms in terms of accuracy, recall, and F1 score, further validating its effectiveness in anomaly detection.

The experiment results show that the anomaly detection capability of the system meets the required standards. The system achieved an accuracy of 0.89, a recall of 0.74, and an F1 score of 0.89, indicating a good balance between precision and recall, as well as demonstrating its effectiveness in detecting anomalies. Additionally, this suggests that the dimensionality-reduced features effectively represent the data, providing a solid foundation for further evaluation.

As shown in Table 2 to assess whether these findings generalize beyond SWaT, we further evaluated the system on the WADI dataset using the same preprocessing pipeline, dimensionality-reduction procedure, and AE-IF settings, with hyperparameters kept fixed to avoid dataset-specific tuning. The WADI experiments yielded consistent trends, indicating that the learned low-dimensional features preserve anomaly-relevant structure and that the framework maintains a favorable precision–recall trade-off across datasets. This cross-dataset evidence underscores the robustness and applicability of the proposed approach to heterogeneous ICS environments and provides a sound basis for subsequent multi-objective evaluation.

### 4.2. Evaluation Methods

In this section, we will assess the accuracy of the scoring. Due to the lack of clear comparison standards, we can only make judgments based on our understanding of the system. Due to the large volume of data, we will only plot the data from the system’s first attack.

**Figure 10:**
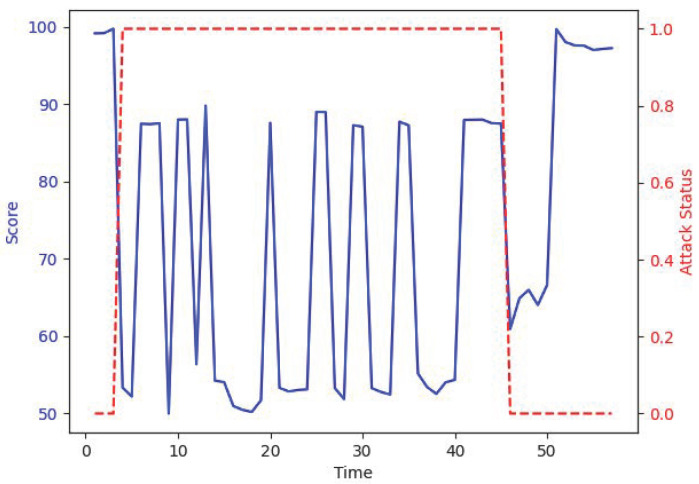
Time vs. score attack.

Figure 10 shows partial scoring data before, during, and after the first attack. The red dashed line indicates whether the system is under attack, with 1 representing an attack and 0 representing no attack. The blue solid line represents the system’s score, with the vertical axis indicating time, and it is labeled sequentially, starting from 1 for simplicity. As can be seen from the figure, the system’s score drops significantly during the attack and exhibits greater fluctuation. This is because, at the time of the attack, certain values in the system were close to their steady-state levels. During the recovery process after the attack, the system’s score gradually improves, but since the system has not yet reached a steady state during recovery, the score remains relatively low for a period of time. Overall, the system’s scores and anomaly detection results behave as expected.

## 5. Conclusions

This paper proposes a scoring system for target evaluation. To address the issue of high target dimensionality, the system first employs an autoencoder for dimensionality reduction. Steady-state data is used to train the autoencoder, and the reduced dimensional data is then used to construct a decision matrix for TOPSIS analysis. Next, post-attack data is processed through the autoencoder for dimensionality reduction, and the reduced data is used to train an Isolation Forest model for anomaly detection. The optimal and worst solutions in the decision matrix are determined based on anomaly distances, and the entropy weight method is used to calculate the weight vector. Finally, the system’s score is calculated using the decision matrix, weight vector, optimal solution, and worst solution.

To validate the effectiveness of the proposed framework, we conducted experiments using the publicly available SWaT dataset, which features a realistic industrial control system environment. This dataset serves as a representative use case for concept validation due to its rich and authentic industrial characteristics. In future work, we plan to extend the evaluation to additional domains, such as IT networks and cloud environments, to further demonstrate the generalizability of our scoring framework.

## Figures and Tables

**Table 1:** Comparison of the different detection methods on the SWaT dataset.

Method	F1-Score	Precision	Recall
SVM [30]	0.796	0.925	0.699
lD-CNN [31]	0.860	0.867	0.854
RNN [30]	0.802	0.982	0.678
KNN [32]	0.350	0.348	0.348
FB [32]	0.360	0.358	0.358
AE [32]	0.520	0.516	0.516
EGAN [32]	0.510	0.406	0.677
AE-IF	0.812	0.745	0.891

**Table 2:** Comparison of the different detection methods on the WADI dataset.

Method	F1-Score	Precision	Recall
PCA	0.250	0.504	0.166
SVM	0.510	0.512	0.512
KNN [32]	0.300	0.299	0.299
FP	0.340	0.336	0.336
AE [32]	0.520	0.520	0.520
EGAN [32]	0.340	0.345	0.345
GAN [32]	0.620	0.538	0.749
AE-IF	0.633	0.587	0.686

## Data Availability

Data available on request from the authors.
